# TMS-Induced Modulation of EEG Functional Connectivity Is Affected by the E-Field Orientation

**DOI:** 10.3390/brainsci13030418

**Published:** 2023-02-28

**Authors:** Giulia Pieramico, Roberto Guidotti, Aino E. Nieminen, Antea D’Andrea, Alessio Basti, Victor H. Souza, Jaakko O. Nieminen, Pantelis Lioumis, Risto J. Ilmoniemi, Gian Luca Romani, Vittorio Pizzella, Laura Marzetti

**Affiliations:** 1 Department of Neuroscience, Imaging and Clinical Sciences, University of Chieti-Pescara, 66100 Chieti, Italy; 2 Department of Neuroscience and Biomedical Engineering, Aalto University School of Science, 02150 Espoo, Finland; 3 BioMag Laboratory, HUS Medical Imaging Center, University of Helsinki and Helsinki University Hospital, 00029 Helsinki, Finland; 4 AMI Centre, Aalto NeuroImaging, Aalto University School of Science, 02150 Espoo, Finland; 5 School of Physiotherapy, Federal University of Juiz de Fora, Juiz de Fora 36036-900, Brazil; 6 Cognitive Brain Research Unit, Department of Psychology and Logopedics, Faculty of Medicine, University of Helsinki, 00100 Helsinki, Finland; 7 Institute for Advanced Biomedical Technologies, University of Chieti-Pescara, 66100 Chieti, Italy

**Keywords:** TMS-EEG, functional connectivity, stimulus orientation

## Abstract

Coregistration of transcranial magnetic stimulation (TMS) and electroencephalography (EEG) allows non-invasive probing of brain circuits: TMS induces brain activation due to the generation of a properly oriented focused electric field (E-field) using a coil placed on a selected position over the scalp, while EEG captures the effects of the stimulation on brain electrical activity. Moreover, the combination of these techniques allows the investigation of several brain properties, including brain functional connectivity. The choice of E-field parameters, such as intensity, orientation, and position, is crucial for eliciting cortex-specific effects. Here, we evaluated whether and how the spatial pattern, i.e., topography and strength of functional connectivity, is modulated by the stimulus orientation. We systematically altered the E-field orientation when stimulating the left pre-supplementary motor area and showed an increase of functional connectivity in areas associated with the primary motor cortex and an E-field orientation-specific modulation of functional connectivity intensity.

## 1. Introduction

Transcranial Magnetic Stimulation (TMS) applies brief magnetic pulses, inducing localized intracranial electric fields (E-fields). The effect is to modulate and elicit brain activity at the targeted area, causing transient alterations of cortical excitability and influencing signal propagation across the cortex [1]. Previous evidence shows that the choice of TMS parameters (e.g., coil position, coil orientation) has a critical impact on the cortical and physiological responses [2,3,4,5,6,7]. Along this line, navigated transcranial magnetic stimulation (nTMS) [8] provides control over these parameters allowing a precise stimulation of cortical areas [9] by exploiting coregistration between the subject’s head position, identified by an infrared camera detecting the optical head trackers and the TMS coil [10,11,12], and individual Magnetic Resonance Imaging (MRI). In this scenario, the adoption of a neuronavigation systems allows the standardization, personalization, and repeatability of the parameter choices based on individual brain structures. Indeed, the introduction of nTMS has allowed the functional mapping of cortical areas as well as the formation of transient functional lesions [13,14].

In addition, nTMS parameters, such as stimulation intensity and timing, are usually adjusted based on peripheral functional responses when available, e.g., when stimulating the motor cortex to observe motor evoked potentials (MEPs) through electromyography [15,16,17,18]. When electroencephalography (EEG) is recorded in a simultaneous coregistration, a more effective nTMS outcome can be obtained by adjusting the stimulation parameters based on the cortico-cortical responses as mapped by nTMS–EEG [19,20]. This strategy is valid for all brain regions even when their stimulation elicits no peripheral response, relying solely on TMS-evoked potentials (TEPs) [21,22,23]. A recent approach using navigated multi-locus TMS (mTMS) [24,25,26], in which a transducer composed of several coils overcomes the slow manual coil adjustment, thus allowing a fast stimulation of different brain locations, enabling to automatically find the optimal position of the E-field in order to maximize peripheral [27] and cortical responses [28]. Indeed, previous studies using mTMS-EEG [28], together with other studies using nTMS–EEG [7,29,30], have shown that changing the E-field orientation causes a different brain response in terms of TEPs.

In this framework, our study investigated, using navigated mTMS and simultaneous EEG, the modulation of the EEG functional connectivity when stimulating the left pre-supplementary motor area (pre-SMA). It is well known, indeed, that the brain is organized in large-scale networks that communicate by synchronizing the activity of oscillating neuronal groups [31,32] to accomplish both integrative and high-order brain functions [33]. The degree of this communication can be assessed through different functional connectivity methods. In particular, methods mainly based on the frequency-specific phase difference between signals of interest [34] are commonly used when fast neurophysiological signals are recorded with EEG.

Specifically, the aim of our study was twofold: firstly, investigate the frequency-specific EEG functional connectivity modulations between the stimulated node and the rest of the brain; secondly, assess whether this functional connectivity modulation could be affected by the induced E-field orientation. For these purposes, we relied on the data collected by Tervo et al. [28], in which EEG was measured while the E-field orientation at the left pre-SMA was electronically changed by navigated mTMS.

## 2. Materials and Methods

### 2.1. Participants

Six healthy participants (two males, one left-handed, aged 22–42) were recruited at the BioMag Laboratory (HUS Medical Imaging Center, Aalto University, University of Helsinki and Helsinki University Hospital). The ethical committee of the Hospital District of Helsinki and Uusimaa (project identification code HUS/1198/2016, date of approval 8 July 2020) approved the study in accordance with the Declaration of Helsinki. Before the experiments, a written informed consent was signed. The data presented here were originally collected by Tervo et al. [28].

### 2.2. The mTMS–EEG Setup

This study takes advantage of the mTMS device, which can electronically generate E-fields in different directions. We stimulated the left pre-SMA, located over the superior frontal gyrus approximately 1–1.5 cm anterior to the vertical anterior commissure line, while simultaneously registering EEG from 64 electrodes with BrainAmp DC amplifiers (Brain Products GmbH, 82205 Gilching, Germany). The identification of the pre-SMA was performed at the single subject level by means of individual structural MRI. Specifically, the MRI was used to reconstruct the 3D brain structure on the neuronavigation system.

**Figure 1:**
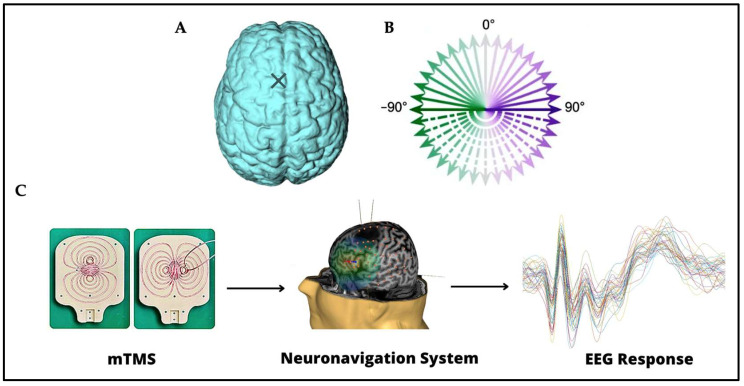
(**A**) The stimulation site on the left pre-SMA is indicated on the brain with a cross. (**B**) The 36 stimulation orientations are represented as green–purple vectors, 0° corresponds to stimulation delivered in the posterior–anterior direction, whereas −180° corresponds to stimulation delivered in the anterior–posterior direction [28]. (**C**) Instruments and techniques used. A 2-coil mTMS transducer, tracked with a neuronavigation system, was used in combination with a 64-channel EEG recording.

See Figure 1 for a schematic representation of the tools used in this study. A monophasic magnetic pulse was applied by a 2-coil mTMS transducer, tracked with an eXimia NBS 3 neuronavigation system (Nexstim Plc, Helsinki, Finland), producing a biphasic E-field in the cortex. The TMS intensity was adjusted when giving the pulses in mediolateral orientation to elicit EEG deflections 15–50 ms after the pulse with 5–10 µV peak-to-peak amplitude when averaging over 20 trials. The signal sampling frequency was 5000 Hz. A low-pass filter with a 1000-Hz cut-off frequency was applied. The auditory response elicited by the click sound provoked by the TMS pulse was masked using earbuds and auditory masking containing white noise and randomly jittering click noise from a recorded coil click [35]. More details about the setup can be found in [28].

### 2.3. Procedure

The original study [28] included a preparatory experiment, Experiment 1, and Experiment 2. We used data from Experiment 1; thus, only procedures related to that part will be included in this section; additional information can be found in [28].

Structural MRI with T1-, fat-suppressed T1-, and T2-weighted sequences were acquired. In this experiment, TMS–EEG was recorded as a function of stimulation orientation by delivering 48 TMS pulses in each of the 36 orientations (full circle at 10° steps; Figure 1B) on the left pre-SMA, divided into 12 blocks for a total of 1728 pulses, with random InterStimulus Interval (ISI) in the range 2.4–2.7 s.

## 3. Data analysis

### 3.1. Preprocessing 

The EEG data were analyzed with MATLAB (version R2020b; The MathWorks, Inc., Natick, MA 01760-2098, USA). Trials were obtained by epoching the data from −600 to 600 ms, where 0 ms is the TMS stimulus onset. The stimulation artifact in the time interval between −2 and 8 ms was discarded and replaced by cubic interpolation. The signal was filtered using a third-order high-pass Butterworth filter (cut-off frequency 1 Hz) in both directions. Trials were visually inspected and rejected if muscle activity or eye blinks were present: on average, 7% of the trials were discarded. Finally, data were baseline corrected by subtracting the average signal in the time interval from −200 to −10 ms from the whole trial and average-referenced. To further attenuate artifacts, the source-estimate-utilizing noise-discarding (SOUND) algorithm [36,37] was applied. More details about the application of the SOUND algorithm can be found in [28]. After the preprocessing step, data from one subject were discarded and not included in the further analysis due to excessive remaining noise in the EEG signals.

### 3.2. Functional Connectivity Estimation

Functional connectivity was calculated between FC1, the electrode closest to the stimulation site, and all the other channels covering the whole head by using the imaginary part of phase locking value (*iPLV*) [38], defined for a single trial and a single frequency band *f* as:

(1) $$iPLV_{m,n}^{tr}\left(f\right)≔\underset{S}{\sum }ℑ(\;e^{i\Delta \theta_{m,n}^{tr}\left(s,f\right)\;})$$

where the sum is computed across time bins *s*, ℑ denotes the imaginary part of a complex-valued number, and *i* is the imaginary unit, e denotes the exponential function, Δθ is the phase difference, *tr* indicates the *iPLV* calculated for a single trial, and *m* and *n* are the indices of the two signals of interest. The signal phase was calculated using the Hilbert transform. We cut the trials into two parts consisting of the pre-stimulus and the post-stimulus intervals ranging from −500 to −10 ms and from 10 to 500 ms, respectively. The analysis was conducted in the theta (4–7 Hz), alpha (8–12 Hz), beta (13–30 Hz), and gamma (38–50 Hz) bands. Signals were filtered in each band separately using a 64-order finite impulse response (FIR) filter with linear phase relying on the Hamming window method. A Yule–Walker autoregressive model with order 30 was used to predict the first and last 364 points of the signal to reduce the edge effects of the Hilbert transform. The signals were Hilbert transformed and then trimmed to the original size. We estimated the instantaneous phase difference between the signal at FC1 and the signals at all other channels to derive single-trial *iPLV* (*iPLV^tr^*) values as in Equation (1). The *iPLV^tr^* value was calculated separately for the pre-stimulus and post-stimulus interval of each trial. To fulfill the first aim of our study, i.e., to assess possible modulations of functional connectivity topographies between pre- and post-stimulus intervals at the group level, we averaged single-trial *iPLV* values across all trials regardless of the E-field orientation. The modulation of functional connectivity was defined as the difference in functional connectivity between FC1 and all other channels in the post-stimulus interval and the same quantity in the pre-stimulus interval. The second aim of our study was to assess, in the channels in which a modulation was observed with the previous analysis (if any), whether the amplitude of functional connectivity (POST-PRE) between FC1 and these channels varies with the E-field orientation. For this purpose, *iPLV^tr^* values in trials in which consecutive E-field orientations in a 20° range were used for the stimulation were considered equivalent among them. This step was necessary since the original protocol was conceived for evoked response analysis of the EEG signal, for which fewer repetitions for each condition (orientation) are needed to obtain reliable information with respect to functional connectivity analysis. In practice, *iPLV^tr^* values for the trials in which the stimulation was delivered at −180° were merged with *iPLV^tr^* values for the trials in which the stimulation was delivered at −170° and macro-orientation is referred to in the following as the −175° macro-orientation. Similarly, for *iPLV^tr^*, values at −160° and −150° stimulus orientation were referred to as the −155° macro-orientation, and so on. This procedure led to 18 different macro-orientations spaced by 20°.

### 3.3. Statistical Assessment 

A one-way analysis of variance (ANOVA) was used to assess the effect of the macro-orientations on the post -minus pre-stimulus *iPLV* values. The model residuals were visually inspected for deviations from normality. Statistical analysis was performed with custom-made scripts written in MATLAB (version R2021b; The MathWorks, Inc., Natick, MA 01760-2098, USA). Specifically, we performed one ANOVA for each hemisphere and for each of the four frequency bands. Thus, a total of eight ANOVAs were computed. For each ANOVA, the levels were equal to the number of macro-orientations, i.e., 18.

In addition, we modeled the functional connectivity modulation as a function of the macro-orientation by a trigonometric polynomial equation, as below:

(2) f(x)=a+bcos(cx)+dsin(cx)

where *x* is the stimulation macro-orientation, and *a*, *b*, *c*, and *d* are the regression coefficients. The goodness-of-fit of the model was measured by *R*-squared (*R*^2^) and tested against the null distribution of *R*-squared values obtained by shuffling data points thousand times (*n* = 1000) with respect to the macro-orientation dimension. The *p*-value was obtained as the proportion of the sample that has an *R*-squared value larger than the non-permuted case.

## 4. Results

### 4.1. iPLV Modulation with Respect to the TMS

**Figure 2:**
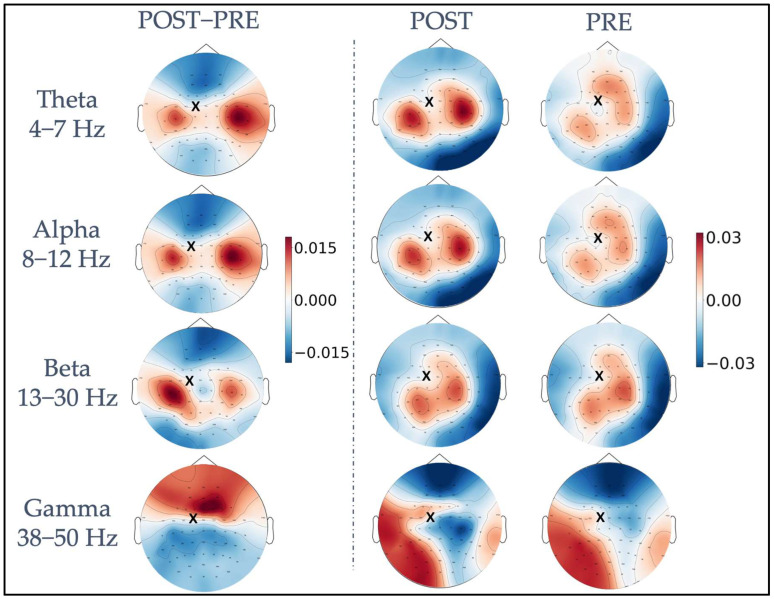
Representation of TMS-induced functional connectivity modulation in the theta, alpha, beta, and gamma frequency bands. The first column shows the difference in *iPLV* between post-stimulus and pre-stimulus conditions, calculated between the FC1 stimulation site (indicated with a black x) and the whole brain. In the second and third columns, post-stimulus *iPLV* and pre-stimulus *iPLV* are represented, respectively.

Figure 2 shows, for each frequency band, *iPLV* values averaged across all trials and subjects in the pre-and post-stimulus intervals, as well as their difference calculated as post- minus pre-stimulus *iPLV* values (leftmost column). A topographically specific functional connectivity pattern can be observed for the theta, alpha, and beta frequency bands between the electrode closest to the stimulated site (FC1, indicated with a black cross in Figure 2) and the rest of the electrodes already before the stimulation (PRE). After the stimulation, in the POST interval, both a change in the topography and an increase in functional connectivity values were observed for the same frequencies. The difference plots (POST−PRE) highlight a topographically specific increase in functional connectivity between FC1 and clusters of electrodes close to bilateral primary motor cortices. Specifically, four left (FC5, FC3, C5, C3) and four right channels (FC4, FC6, C4, C6) were considered as “channels of interest”. Of note, in the theta and alpha bands, the connectivity was higher in the channels close to the primary motor cortex in the right hemisphere, whereas in the beta band, the connectivity was higher in channels close to the primary motor cortex in the left hemisphere. Visual inspection of gamma band *iPLV* modulation topography revealed a widespread pattern, indicating that the modulation is not related to functional connectivity within the motor network.

### 4.2. Functional Connectivity Depends on the Stimulus Orientation

The one-way ANOVAs revealed significant dependency on the macro-orientation of the modulations of functional connectivity between FC1 and left channels close to the motor cortices in theta (*F*(1,17) = 3.02, *p* < 10^−5^), alpha (*F*(1,17) = 3.03, *p* < 10^−5^), and beta bands (*F*(1,17) = 2.14, *p* < 0.004). Similar results were found for the modulations of functional connectivity between FC1 and right channels in theta (*F*(1,17) = 2.96, *p* < 10^−5^), alpha (*F*(1,17) = 3.14, *p* < 10^−5^), and beta band (*F*(1,17) = 3.67, *p* < 10^−7^).

**Figure 3:**
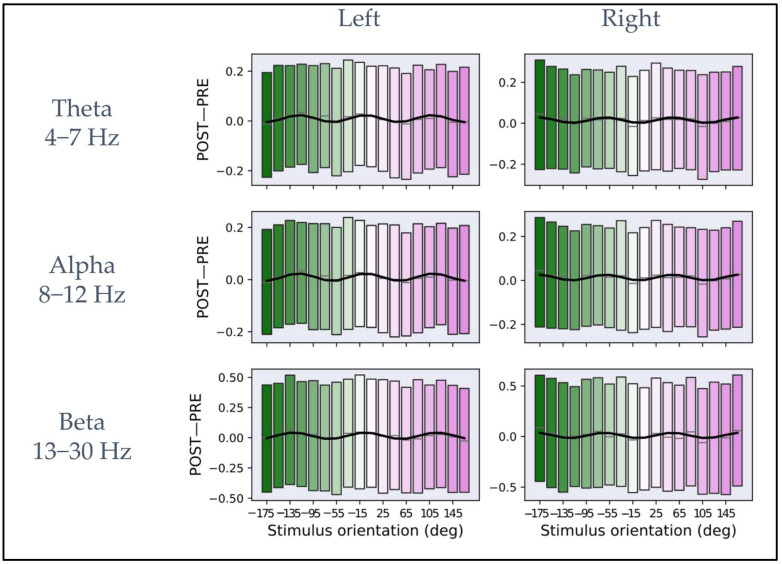
Plots of *iPLV* differences between post- and pre-stimulus intervals averaged across macro-orientations of 20° for theta, alpha, and beta frequency bands. In the (**left column**), the plots represent the average as a function of the macro-orientation for the channels close to the left primary motor cortex for each frequency band. In the (**right column**), the plots represent the mean as a function of orientation for the channels close to the right primary motor cortex for each frequency band. In each plot, the rectangular boxes represent the data from the 25th to the 75th percentile with the same reference colors as Figure 1B. The lines inside the rectangular boxes represent the mean (gray line). The data are fitted on the mean (solid black curve) with the model described in Equation (2).

Figure 3 shows the dependency of *iPLV* modulation (POST−PRE) from the macro-orientations defined in Section 3.2 for the theta, alpha, and beta bands and the channel clusters close to the bilateral primary motor cortices. The black line inside each rectangular box represents the mean across all subjects and trials corresponding to each macro-orientation. The lower and upper boundaries of the rectangular boxes represent the data from the 25th to the 75th percentile for each macro-orientation and color coding, as in Figure 1B. The macro-orientation-specific *iPLV* modulation (indicated by the blue curves in all panels of Figure 3) was obtained by fitting the mean calculated across all subjects and the trials in a macro-orientation group and to the model described in Equation (2). Table 1 shows the results for the fit parameters for each frequency band and for the two-channel clusters.

The *R*^2^ was calculated for estimating the goodness-of-fit between the data and the model in each frequency band, and its statistical significance was assessed by comparison to the null distribution obtained by shuffling the data points. The *R*^2^ values and their statistical significance are: in the theta (left: *R*^2^ = 0.60, *p* < 0.001; permutation test, *n* = 1000, right: *R*^2^ = 0.43, *p* < 0.001; permutation test, *n* = 1000), alpha (left: *R*^2^ = 0.66, *p* < 0.001; permutation test, *n* = 1000, right: *R*^2^ = 0.42, *p* < 0.001; permutation test, *n* = 1000), and beta band (left: *R*^2^ = 0.61, *p* < 0.001; permutation test, *n* = 1000, right: *R*^2^ = 0.41, *p* < 0.001; permutation test, *n* = 1000).

For all bands, the same qualitative behavior across the two hemispheres can be observed. Specifically, three maxima and three minima can be observed on the left and right primary motor cortex with reverse “polarity” between hemispheres. Interestingly, both for left pre-SMA and left motor cortex and for left pre-SMA and right motor cortex connectivity, an average periodicity of about 120°, estimated from the fit parameters, was observed in the dependency from the E-field orientation. In the Supplementary Material, Figures S1–S5 show the average of *iPLV* modulation (POST−PRE) in the macro-orientation groups for theta, alpha, and beta bands for each individual subject. 

## 5. Discussion

We investigated the role of the TMS E-field orientation in the modulation of EEG functional connectivity. For this purpose, we used the novel mTMS device with the rotational transducer (two-coil version [25]) in conjunction with a neuronavigation system to precisely identify the location and intensity of the stimulation and quickly and effectively adjust the stimulus orientation. Importantly, the possibility of electronically changing the stimulus orientation allows a more dense and more precise sampling of the orientation space with respect to manual coil rotation.

We demonstrated that the stimulation of the left pre-SMA induced an increase of functional connectivity between the stimulated site and left and right primary motor cortices in the theta, alpha and beta frequency bands. This finding is in line with several studies showing the role of the pre-SMA in the motor network since this area is crucial for the initiation and inhibition of voluntary action, control of sequential movements, and cognitive control of actions [39,40,41,42]. In addition, pre-SMA plays a key role in complex motor behavior, including self–initiated activity, generation of action sequences and motor planning and learning [43,44]. This has also been confirmed in studies performed with TMS in the context of switching and stopping behavior [45,46]. Indeed, the posterior portion of the pre-SMA has been shown to feature connections with motor areas in monkeys [47]. Moreover, the frequency bands in which we observed the connectivity modulation are usually involved in the modulation of attention or working memory but are also sensitive to higher motor control functions, i.e., functions regulated by pre-SMA [48,49]. More specifically, we observed a predominant increase in functional connectivity between the left pre-SMA and right motor cortex in the theta and alpha bands. Conversely, in the beta frequency band, we observed a higher modulation of functional connectivity between the left pre-SMA and left motor cortex. Possibly, this hemispheric difference in the different frequency bands (theta–alpha versus beta) is due to a different functional role of the induced increase in functional connectivity. Specifically, contralateral functional connectivity between the left pre-SMA and right motor cortex is higher in the theta and alpha frequency bands due to a possible inhibitory functional role of this connection. Conversely, functional connectivity between the left pre-SMA and ipsilateral left motor cortex is higher in the beta frequency band due to a possible excitatory functional role of this connection.

Furthermore, we showed that functional connectivity was modulated by the E-field orientation. Specifically, we found that when the coil was oriented along the −175° macro-orientation, i.e., when the E-field was oriented close to the anterior–posterior direction, the maximal increase in functional connectivity between the left pre-SMA and the right motor cortex was observed. Conversely, when the coil was oriented along the −115° macro-orientation, the maximal increase in functional connectivity between the left pre-SMA and the left motor cortex was observed. Several studies showed that the direction of the TMS coil influences brain responses, and there is already ample evidence of the importance of coil orientation for the stimulation of the primary motor cortex [25,29,30,50,51,52]. Less is known for the stimulation of SMA. In general, the effects of different E-field orientations on a specific brain area depend on the cellular composition of this area. Indeed, several lines of evidence pointed out the structural and excitability heterogeneity of pre-SMA [47,53], factors that might justify the different responses of the same region to different stimulus orientations. Indeed, a recent study by Casula et al. [29] demonstrated that by stimulating pre-SMA, different populations of neurons could be activated by varying coil orientation and stimulation intensities. The authors showed that brain oscillatory response modulations in the theta–alpha range are higher when E-field points anteriorly compared to any other tested orientation, while the same and other coil orientations showed higher brain oscillatory response modulations in the beta band. These results for spectral perturbation do not strictly match ours in terms of the coil orientations at which maximal effects are observed, possibly because in [29], the observed spectral perturbation is calculated for channels close to the stimulation site, thus capturing TMS-induced modulation of local induced activity, whereas our results capture long-range phase coupling of oscillatory brain activity.

Moreover, our results show a periodicity of about 120° in the modulation of functional connectivity with the E-field orientation. A similar modulation is visible, although to a minor extent, also at the single subject level, as illustrated in Figures S1–S5 in the Supplementary Material. Possibly, this periodicity is related to the stimulation targeting differently oriented neuronal populations in the pre-SMA/SMA complex.

This study presents some limitations. First, the number of trials in each stimulation orientation is low for functional connectivity estimation. To overcome this limitation, we downsampled the orientation space by a factor of two; nevertheless, a protocol with more trials for each orientation would be desirable for the specific purpose of evaluating functional connectivity modulations. Second, the number of subjects that underwent this experimental protocol is limited, and a more extensive investigation could allow the future to derive even more specific results on the dependency of functional connectivity on stimulus orientation.

## 6. Conclusions

We showed that the functional connectivity between the left pre-SMA and the right and left primary motor cortices could be modified by applying TMS on the left pre-SMA. The E-field orientation at the stimulation site induces different modulations of the functional connectivity in the theta, alpha, and beta frequency bands, showing that stimulation orientation is a crucial determinant of the modulation of brain functional connectivity. Our results point towards the possibility of relying on orientation-specific stimulation to modulate the connectivity of brain networks, which can eventually be used as feedback for closed-loop brain stimulation [28].

## Figures and Tables

**Table 1:** Fit parameters as defined by Equation (2).

Frequency Bands	a	b	c	d
Theta Left	0.009	−0.014	1.100	−0.001
Theta Right	0.015	0.013	1.089	−0.002
Alpha Left	0.009	−0.014	1.083	0.003
Alpha Right	0.013	0.013	1.086	−0.002
Beta Left	0.016	−0.019	1.022	0.019
Beta Right	0.010	0.025	1.072	−0.009

## Data Availability

According to the ethical permissions statement of the Aalto University, experimenters are not allowed to make physiological or anatomical data publicly available. However, the data can be accessed upon reasonable request as long as the confidentiality requirements are strictly followed.

## Supplementary Materials

The following supporting information can be downloaded at: https://www.mdpi.com/article/10.3390/brainsci13030418/s1, Figure S1: Plots of *iPLV* differences between post- and pre-stimulus intervals on subject 3, averaged across macro-orientations of 20° for theta, alpha and beta frequency bands. In the first column, the plots represent the average as a function of the macro-orientation for the channels close to the left primary motor cortex for each frequency band. In the second column, the plots represent the mean as a function of orientation for the channels close to the right primary motor cortex for each frequency band. Figure S2: Plots of *iPLV* differences between post- and pre-stimulus intervals on subject 4, averaged across macro-orientations of 20° for theta, alpha and beta frequency bands. In the first column, the plots represent the average as a function of the macro-orientation for the channels close to the left primary motor cortex for each frequency band. In the second column, the plots represent the mean as a function of orientation for the channels close to the right primary motor cortex for each frequency band. Figure S3: Plots of *iPLV* differences between post- and pre-stimulus intervals on subject 5, averaged across macro-orientations of 20° for theta, alpha and beta frequency bands. In the first column, the plots represent the average as a function of the macro-orientation for the channels close to the left primary motor cortex for each frequency band. In the second column, the plots represent the mean as a function of orientation for the channels close to the right primary motor cortex for each frequency band. Figure S4: Plots of *iPLV* differences between post- and pre-stimulus intervals on subject 7, averaged across macro-orientations of 20° for theta, alpha and beta frequency bands. In the first column, the plots represent the average as a function of the macro-orientation for the channels close to the left primary motor cortex for each frequency band. In the second column, the plots represent the mean as a function of orientation for the channels close to the right primary motor cortex for each frequency band. Figure S5: Plots of *iPLV* differences between post- and pre-stimulus intervals on subject 8, averaged across macro-orientations of 20° for theta, alpha and beta frequency bands. In the first column, the plots represent the average as a function of the macro-orientation for the channels close to the left primary motor cortex for each frequency band. In the second column, the plots represent the mean as a function of orientation for the channels close to the right primary motor cortex for each frequency band.
