# Supplementary material for: TMS-Induced Modulation of EEG Functional Connectivity Is Affected by the E-Field Orientation
Source: Brain Sci. 2023 Feb 28;13(3):418. doi: 10.3390/brainsci13030418 (PMC10046030; doi:10.3390/brainsci13030418)
Supplement: Supplementary file 1 [file brainsci-13-00418-s001.zip › brainsci-2223053-supplementary.pdf]

Supplementary Materials

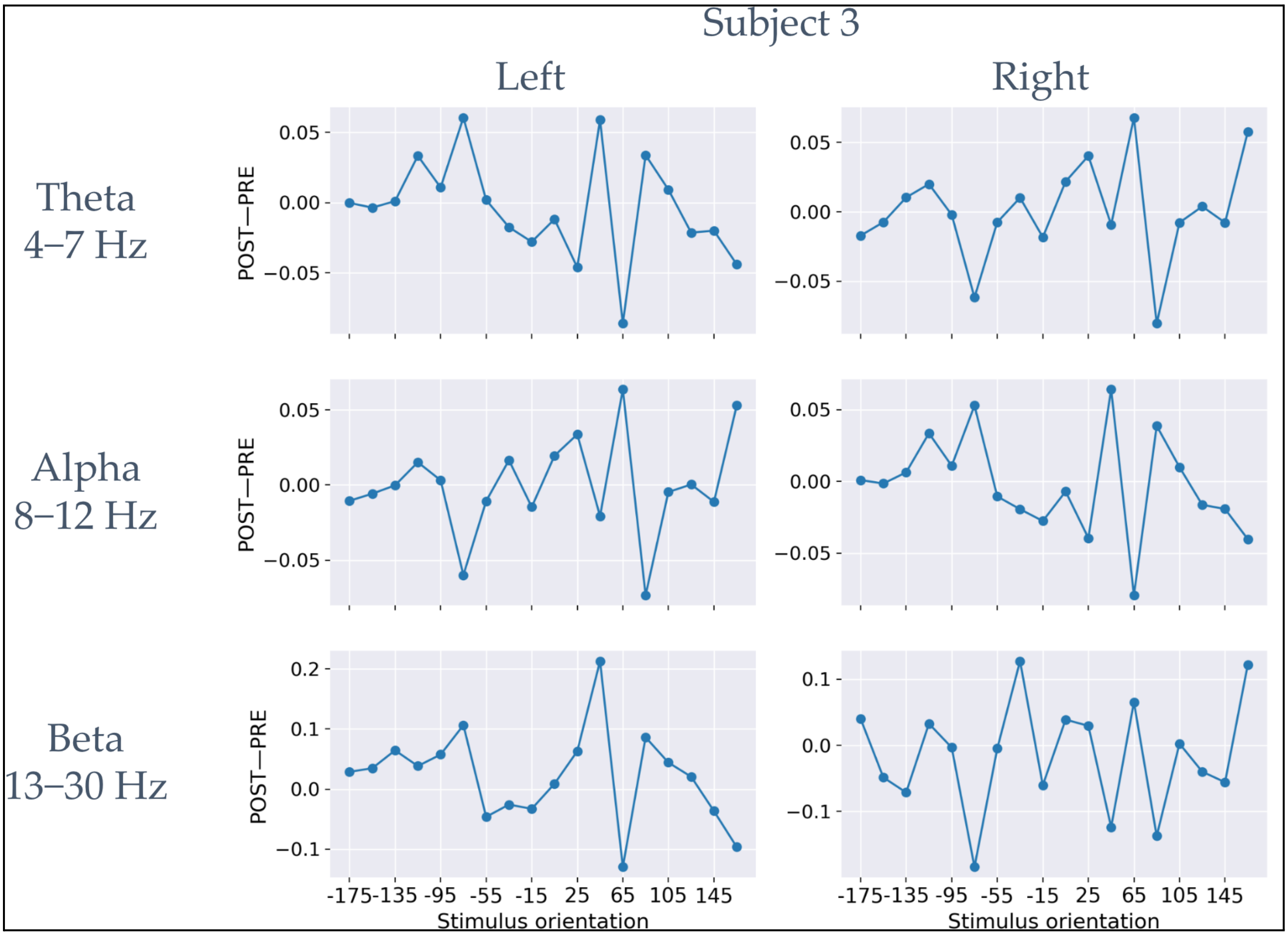

Figure S1| Plots of iPLV differences between post- and pre-stimulus intervals on subject 3, averaged across macro-orientations of 20° for theta, alpha and beta frequency bands. In the first column, the plots represent the average as a function of the macro-orientation for the channels close to the left primary motor cortex for each frequency band. In the second column, the plots represent the mean as a function of orientation for the channels close to the right primary motor cortex for each frequency band.

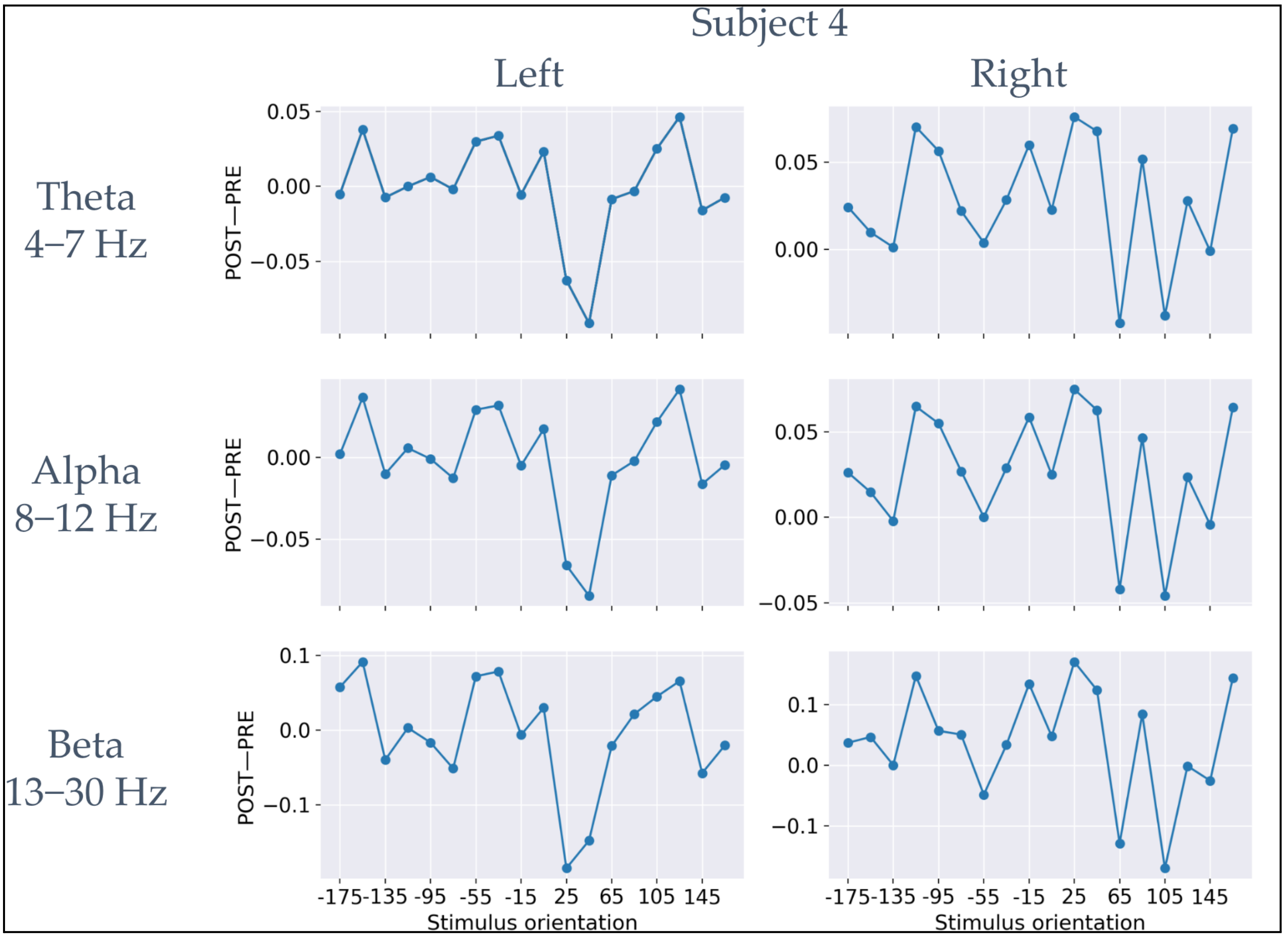

Figure S2| Plots of iPLV differences between post- and pre-stimulus intervals on subject 4, averaged across macro-orientations of 20° for theta, alpha and beta frequency bands. In the first column, the plots represent the average as a function of the macro-orientation for the channels close to the left primary motor cortex for each frequency band. In the second column, the plots represent the mean as a function of orientation for the channels close to the right primary motor cortex for each frequency band.

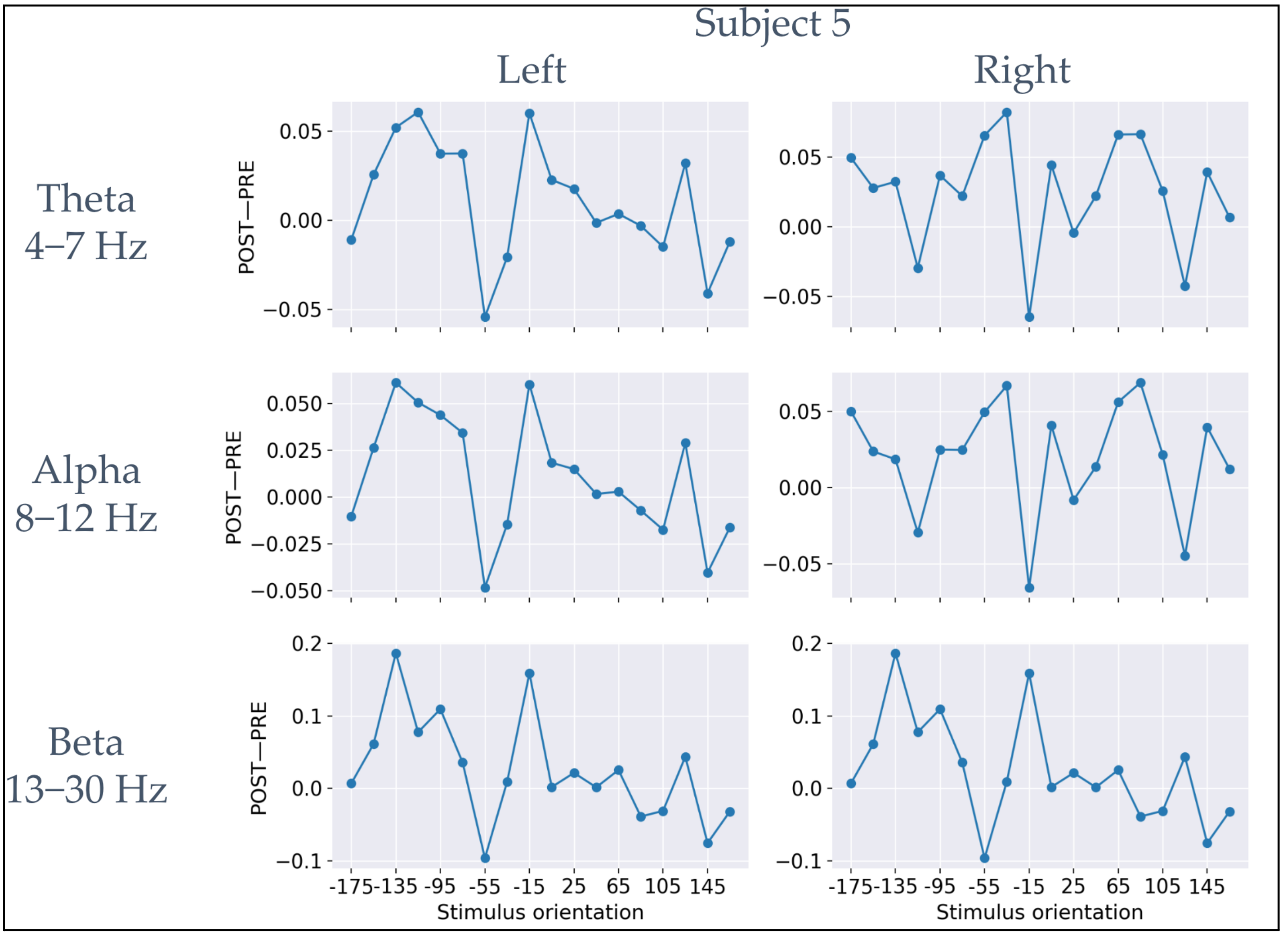

Figure S3| Plots of iPLV differences between post- and pre-stimulus intervals on subject 5, averaged across macro-orientations of 20° for theta, alpha and beta frequency bands. In the first column, the plots represent the average as a function of the macro-orientation for the channels close to the left primary motor cortex for each frequency band. In the second column, the plots represent the mean as a function of orientation for the channels close to the right primary motor cortex for each frequency band.

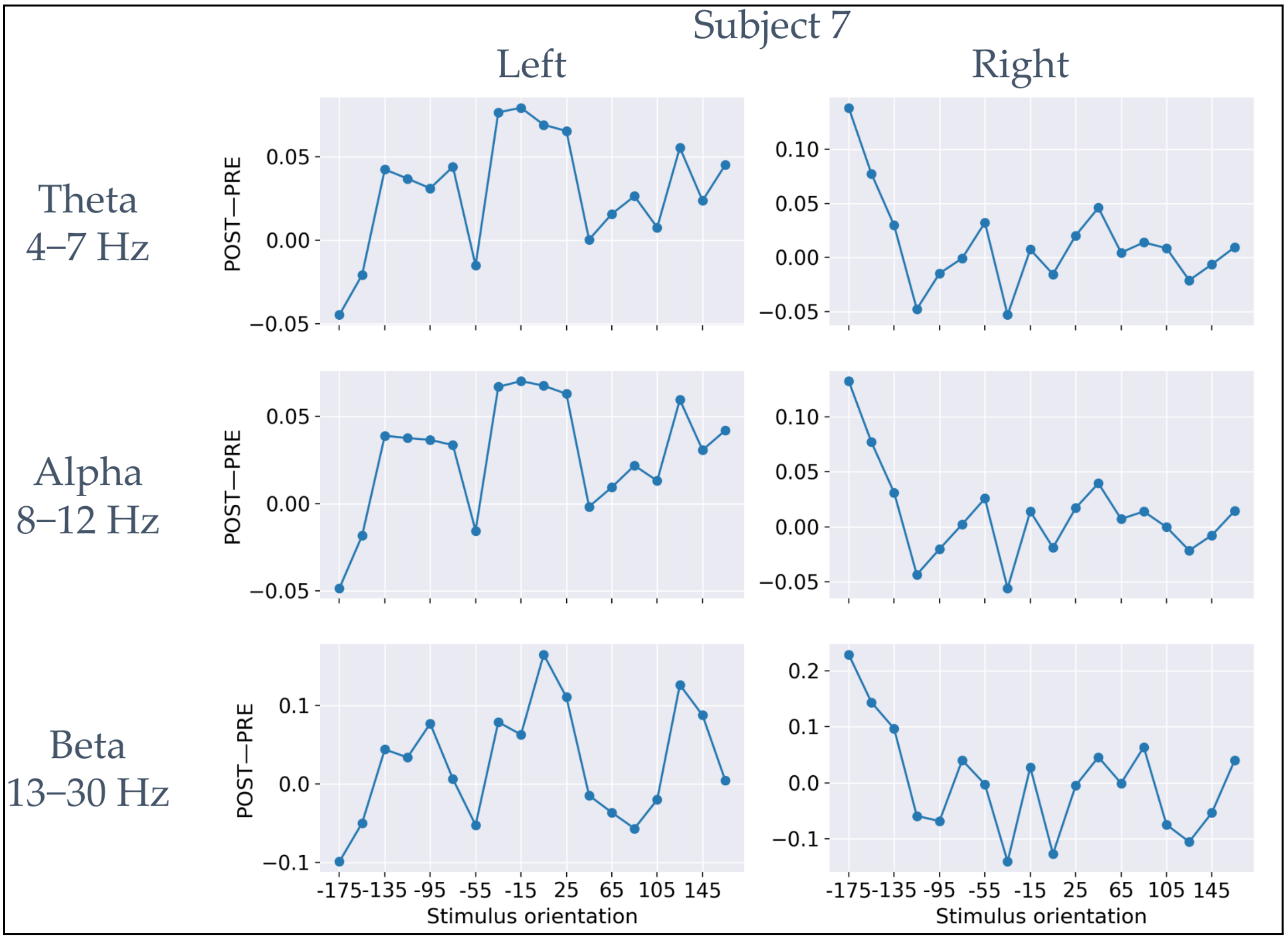

Figure S4| Plots of iPLV differences between post- and pre-stimulus intervals on subject 7, averaged across macro-orientations of 20° for theta, alpha and beta frequency bands. In the first column, the plots represent the average as a function of the macro-orientation for the channels close to the left primary motor cortex for each frequency band. In the second column, the plots represent the mean as a function of orientation for the channels close to the right primary motor cortex for each frequency band.

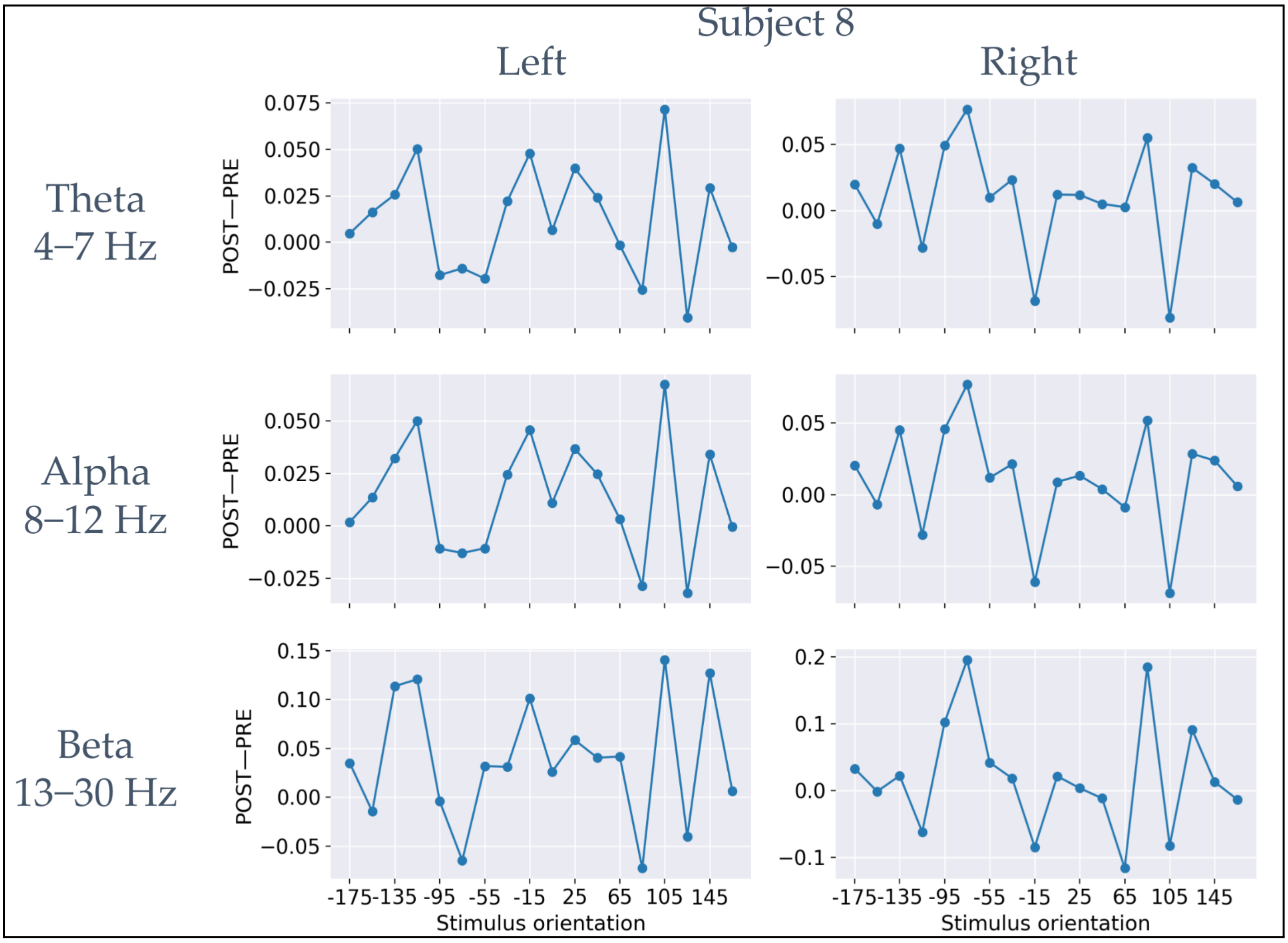

Figure S5| Plots of iPLV differences between post- and pre-stimulus intervals on subject 8, averaged across macro-orientations of 20° for theta, alpha and beta frequency bands. In the first column, the plots represent the average as a function of the macro-orientation for the channels close to the left primary motor cortex for each frequency band. In the second column, the plots represent the mean as a function of orientation for the channels close to the right primary motor cortex for each frequency band.
